# Serological Response to COVID-19 and Its Association With Measles-Rubella (MR)-Containing Vaccines

**DOI:** 10.7759/cureus.39671

**Published:** 2023-05-29

**Authors:** Jahnavi Shrivastava, Manish Narang, Rafat S Ahmed, Shukla Das, Sunil Gomber

**Affiliations:** 1 Pediatrics, University College of Medical Sciences (University of Delhi) and Guru Tegh Bahadur Hospital, Delhi, IND; 2 Biochemistry, University College of Medical Sciences (University of Delhi) and Guru Tegh Bahadur Hospital, Delhi, IND; 3 Microbiology, University College of Medical Sciences (University of Delhi) and Guru Tegh Bahadur Hospital, Delhi, IND

**Keywords:** children, antibody response, rubella vaccine, measles vaccine, immune response, vaccine efficacy, immunogenicity, sars-cov-2

## Abstract

Background and objectives: Epidemiological studies suggest that coronavirus disease 2019 (COVID-19) has a less severe disease course and a more favorable prognosis among children. Childhood vaccines and heterologous immunity have been suggested as reasons for this. Additionally, the structural similarity between the measles, rubella, and severe acute respiratory syndrome coronavirus 2 (SARS-CoV-2) virus particles may affect immune responses. The objective of this study was to compare COVID-19 antibody titers and disease severity between measles-rubella (MR) vaccinated and unvaccinated children. Additionally, we aimed to evaluate and compare the antibody response in recipients of a single dose and two doses of the MR vaccine.

Methods: The study was prospective and comparative and included 90 COVID-19-positive children aged nine months to 12 years. The study was registered under the clinical trials registry of India (CTRI/2021/01/030363). COVID-19 antibody titers were measured at two weeks, six weeks, and 12 weeks, along with the assessment of MR antibody titers. COVID-19 antibody titers and disease severity were compared between MR-vaccinated and MR-unvaccinated children. The comparison of COVID-19 antibody titers between recipients of a single dose and two doses of MR vaccine was also conducted.

Results: The results showed significantly higher median COVID-19 antibody titers at all time points during follow-up in the MR-vaccinated group (P<0.05). However, the two groups had no significant difference in the disease severity. Moreover, there was no difference in the antibody titers of MR one dose and two dose recipients.

Conclusion: Exposure to even a single dose of MR-containing vaccine enhances the antibody response against COVID-19. However, randomized trials are necessary to further explore this subject.

## Introduction

COVID-19 has been observed to have a less severe impact on children than adults, partly due to the induction of heterologous immunity against SARS-CoV-2 by various childhood vaccines [1]. The Bacille Calmette Guerin (BCG), measles mumps rubella vaccine (MMR), and oral polio vaccine (OPV) have been hypothesised to modulate the course of COVID-19 by priming immune cells to combat infection through producing epigenetic changes [2]. Among these, measles and rubella containing vaccines (MRCVs) have emerged as promising candidates due to their homology with the spike (S) glycoprotein of the SARS-CoV-2 virus, the most promising vaccine target for COVID-19 [3,4]. Previous research on measles vaccination has shown a reduction in morbidity and mortality due to respiratory pathogens other than measles virus, particularly in children from low and middle-income countries [5-7].

Measles and rubella vaccines are included in the immunisation programs of 173 member states, making their use against COVID-19 more feasible [8]. While descriptive studies have been conducted, promising protection against severe COVID-19 in MMR recipients, objective data on the subject remains scarce [9,10]. This study was designed to explore the impact of MR-containing vaccines on the antibody response and course of disease in COVID-19 infected children.

Our study aimed to compare COVID-19 antibody titres between MR-vaccinated and MR-unvaccinated children up to 12 weeks post-infection as its primary objective. The secondary objective was to compare disease severity between MR-vaccinated and unvaccinated children. Moreover, we sought to determine whether there was any difference in the serological response between those who received one dose and two doses of MR-containing vaccine.

## Materials and methods

This prospective comparative study was conducted from December 2020 to April 2022 in the Pediatrics department of a tertiary care hospital in northern India. Ethical clearance was obtained from the Institutional Ethics Committee-Human Research. The study was registered under the Clinical Trials Registry of India (CTRI/2021/01/030363).

Participant selection

Children, aged nine months to 12 years, who tested positive for COVID-19 infection by rapid antigen test or RT-PCR were enrolled into our study. Children with past infection due to measles or rubella, immunodeficiency states (both primary and secondary), any known chronic disease (congenital heart disease, respiratory diseases that cause pulmonary function impairment, hypothyroidism, insulin-dependent diabetes mellitus {IDDM}), recipients of blood transfusion within last three months, severe malnutrition or malignancy were excluded from the study. Written informed consent was obtained from a family member or a surrogate for participation in the study before any study-related procedure was performed. Assent was obtained from the participating children >7 years of age.

Sample collection and follow-up

Children testing positive for COVID-19 were sorted into two groups based on their MR- vaccination status which was ascertained using immunization records and by recall method. The baseline anthropometry was recorded along with other demographic parameters on the case record form. The duration and nature of symptoms, requirement of hospitalization and oxygen support, and details of complications, if any, were noted for each child. They were then classified into asymptomatic, mild, moderate, severe, or critically ill, based on the Ministry of Health and Family Welfare, India, guidelines for COVID-19 case management [11].

COVID-19 antibody titers were estimated at two weeks, six weeks, and 12 weeks from the date of the positive test by a chemiluminescence assay-based immunoglobulin (Ig)G antibody test. Additionally, measles and rubella antibody titers were done at six weeks.

Sample size

90 children were recruited into this pilot study, out of which 64 were MR-vaccinated and 26 were unvaccinated. We ensured that these children differed only in the receipt of an MR-containing vaccine and had received the remaining vaccines as per schedule to avoid bias.

Statistical analysis

Statistical analysis was undertaken as per the intention to treat analysis. Qualitative variables were analyzed using the Chi-square test and qualitative variables were analyzed using Fischer exact test. Mann-Whitney U test was employed to compare the antibody titers between vaccinated and unvaccinated groups. The Spearman correlation coefficient was calculated to derive the association between measles, rubella, and COVID-19 antibody titers. SPSS software, version 20.0 (IBM Corp., Armonk, NY) was used for analysis.

## Results

Enrolment and baseline characteristics

**Figure 1:**
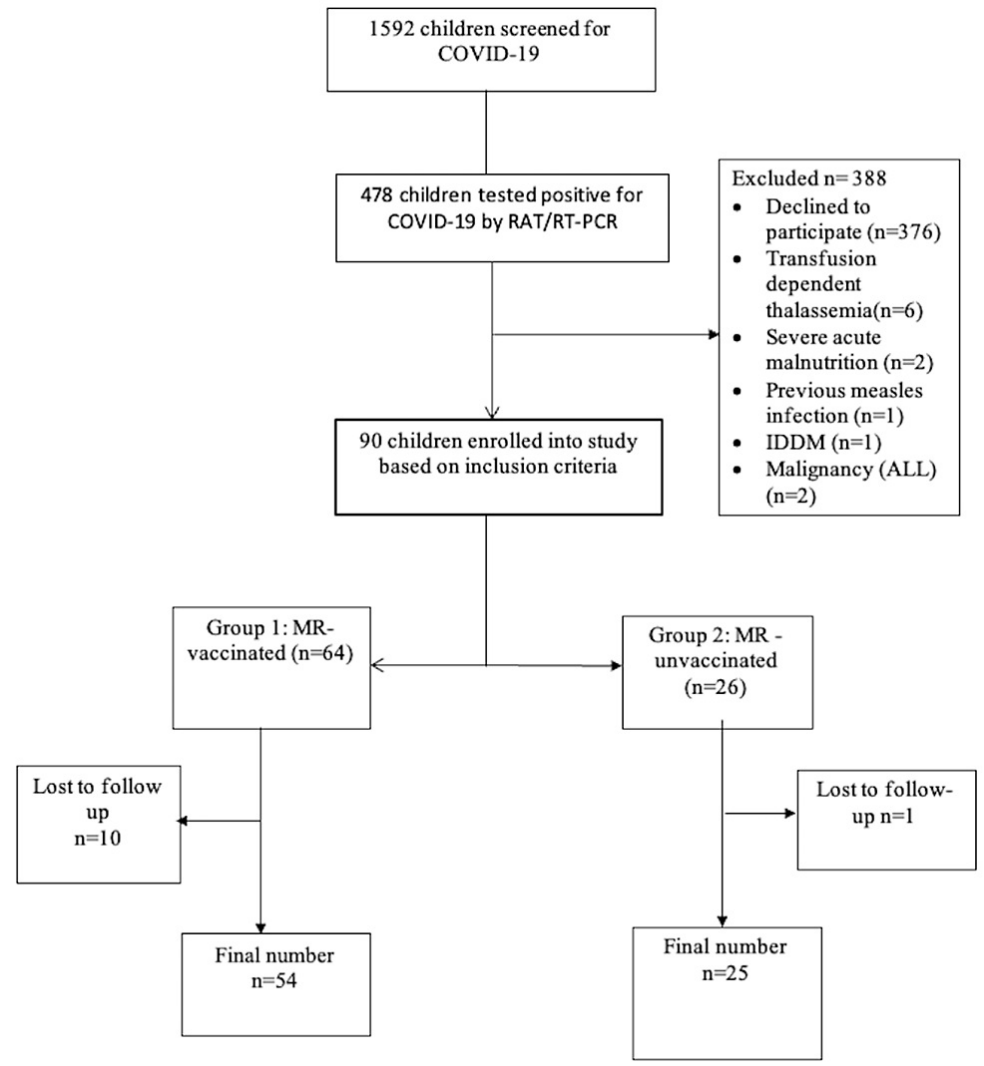
Study flowchart RAT: Rapid antigen test; RT-PCR: Reverse transcriptase polymerase chain reaction; IDDM: Insulin-dependent diabetes mellitus; ALL: Acute lymphoblastic leukaemia; MR: Measles-Rubella

The study enrolled 90 children, 44 of whom were male, with a median age of 85 months (interquartile range {IQR} 39.5-115.5). Figure 1 shows the study's participant flow. The age (in months) of both the MR-vaccinated and unvaccinated groups was comparable, with a median (IQR) of 85 (41.4-116.5) versus 90 (30.75-114.75), respectively (P>0.05). The gender distribution (as a percentage) was also similar in both groups, indicating that the study groups were homogeneous in terms of age and gender (Table 1).

**Table 1:** Demographic profile of the study population MR: Measles-Rubella

	MR vaccinated (n=64)	MR unvaccinated (n=26)	Total population (n=90)
9m-1yr (n{%})	2 (3.1%)	2 (7.7%)	4 (4.4%)
1-5 years (n{%})	21 (32.8%)	7 (26.9%)	28 (31.1%)
5-12 years (n{%})	41 (64.1%)	17 (65.4%)	58 (64.4%)
Age (months) Median (IQR)	85 (41.5-116.5)	90 (30.75-114.75)	85 (39.5-115.5)
Male (n{%})	34 (53.1%)	10 (38.5%)	44 (48.9%)
Female (n{%})	30 (46.9%)	16 (61.5%)	46 (51.1%)

MR vaccine efficacy

Compared to the unvaccinated group, MR-vaccinated children had a significantly higher median (IQR) antibody titre value two weeks after vaccination (8.42 S/CO {2.25-19.40} vs. 1.64 S/CO {0.89-11.72}; P<0.01) (S/CO: standard cutoff). Vaccinated children had significantly higher titres at six weeks (4.97 S/CO {1.21-15.14} vs. 1.22 S/CO {0.65-4.30}; P<0.01) and twelve weeks of follow-up (2.44 S/CO {0.97-6.15} vs. 0.98 S/CO {0.62-1.98}; P=0.02) (Table 2).

**Table 2:** Comparison of antibody titres and clinical severity between MR-vaccinated and unvaccinated children MR: Measles-Rubella; COVID: Coronavirus disease

	MR vaccinated group (n=64)	MR unvaccinated group (n=26)	P value
COVID antibody titers at 2 weeks	8.42 (2.25-19.40)	1.64 (0.89-11.72)	<0.01
COVID antibody titers at 6 weeks	4.97 (1.21-15.14)	1.22 (0.65-4.30)	<0.01
COVID antibody titers at 12 weeks	2.44 (0.97-6.15)	0.98 (0.62-1.98)	0.02
Asymptomatic category	21 (32.8%)	8 (30.8%)	0.85
Symptomatic category	43 (67.2%)	18 (69.2%)	

Disease severity

Of the vaccinated group, 32.8% and of the unvaccinated group, 30.8% belonged to the "asymptomatic" category of disease, while 64.1% of the vaccinated group and 69.2% of the unvaccinated group reported a mild course. One child had COVID pneumonia (moderate category), and one had acute respiratory distress syndrome (ARDS) and shock (critically ill), both of whom were in the MR-vaccinated group. Due to a low number of cases in the "moderate," "severe," and "critically ill" categories, the data had to be dichotomised for analysis (Table 2). The number of children (as a percentage) in the MR-vaccinated and unvaccinated groups did not differ significantly in terms of disease severity (32.8% vs. 30.8% for asymptomatic group and 67.2% vs. 69.2% for the symptomatic category; P=0.85).

Single vs. two doses of MR vaccine

The median (IQR) COVID antibody titres of the recipients of a single dose of MR-containing vaccine and two doses were not significantly different at the two-week follow-up (14.07 {2.71-24.37} vs. 8.30 {2.20-17.78}; P=0.44). Similar results were obtained at the six-week visit (6.73 {1.12-20.59} vs. 4.89 {1.25-12.63}; P=0.54) and at the twelve-week follow-up (2.85 {0.89-15.48} vs. 2.44 {0.98-5.01}; P=0.46) (Table 3).

**Table 3:** Comparison of COVID-19 antibody titres between one dose and two doses of MR-containing vaccine MR: Measles-Rubella

Time after COVID-19 positive test	COVID antibody titers in MR vaccinated (1 dose) group (n=10)	COVID antibody titers in MR vaccinated (2 doses) group (n=54)	P value
2 weeks	14.07 (2.71-24.37)	8.30 (2.20-17.78)	0.44
6 weeks	6.73 (1.12-20.59)	4.89 (1.25-12.63)	0.54
12 weeks	2.85 (0.89-15.48)	2.44 (0.98-5.01)	0.46

**Figure 2:**
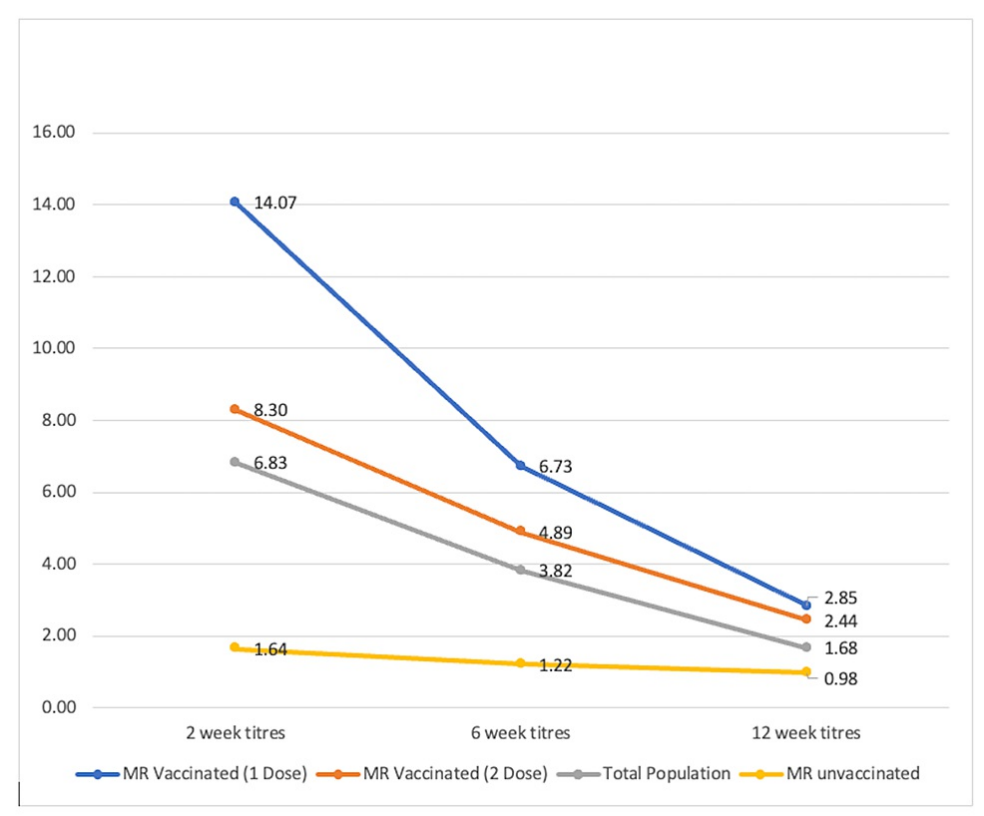
Trend of COVID-19 antibodies till twelve weeks post-infection MR: Measles-Rubella

Our study has provided additional important information. We observed that 16 children (17.8%) did not develop detectable levels of antibodies throughout the study. Furthermore, 30.8% of the unvaccinated children in our study were found to be seronegative, while only 12.5% of the vaccinated group showed the same result (P value 0.04). Additionally, our study found a significant and rapid decline in antibody levels during the follow-up period (intra-group P value for 2-12 weeks <0.0001) as demonstrated in Figure 2. Finally, we also found a moderate positive correlation between antibody levels against measles and rubella with those against COVID-19 (r=0.51-0.64, P value <0.001), for the 2-12 week period.

## Discussion

The study discussed in this article examined the serological response to COVID-19 and its association with MR-containing vaccines. The results showed that individuals who received MR-containing vaccines had higher antibody titres compared to those who were unvaccinated, and the number of vaccine doses did not impact the immune response. Disease severity was not different between the two groups. The findings of this study add to the growing body of evidence suggesting that prior vaccination with the measles-rubella vaccine may confer some level of protection against COVID-19 [12]. This is consistent with the concept of heterologous immunity, which proposes that prior exposure to one pathogen can elicit an immune response that provides some level of protection against a different, unrelated pathogen. The underlying mechanism for this is thought to involve the activation of memory T cells and B cells, which can cross-react with antigens from other pathogens and provide a more rapid and effective response upon subsequent exposure. Interestingly, the results of this study suggest that even a single dose of the measles-rubella vaccine may be sufficient to boost the immune response to subsequent COVID-19 exposure. This has important implications for vaccine efficacy, as it suggests that individuals who have received the MMR vaccine may be less likely to experience vaccine failure if they subsequently receive a COVID-19 vaccine. However, it is important to note that this finding should be confirmed by independent clinical trials with larger sample sizes and longer follow-up periods.

The findings of this study support a previous study by Hassani et al. which reported higher anti-measles IgG in COVID-19 patients and a positive correlation between measles and COVID-19 nucleoprotein-specific IgG antibodies [13]. However, a study by Kandeil et al. on BALB/c mice produced different results, possibly due to the use of a different strain of MMR vaccine and different antibody estimation kits [14]. Lundberg et al. in their survey-based study concluded that recipients of two doses of MR-containing vaccine had higher protection rates and consequently, lower probability of testing positive for COVID-19 [15].

It is possible that the current study differs from previous research due to the limited number of cases included in the single-dose MR vaccine group. This may have impacted the antibody titres, potentially resulting in a false lack of association. In terms of disease severity, the current study is consistent with a previous study by Gujar et al., which reported fewer symptomatic cases in the MR-vaccinated group compared to the unvaccinated group [16]. However, the current study may have been limited by the recruitment of mostly asymptomatic and mild cases, and further studies with larger sample sizes and longer follow-up periods are needed.

The current study also identified a higher number of serological non-responders in the MR-unvaccinated group, which may have implications for vaccine effectiveness. A study by Liu et al. found that younger age and lower viral loads were associated with poor immune response, and non-seroconverters may have a similar poor response to vaccination [17]. Additionally, the current study highlights the relevance of studying the impact of heterologous immunity on the disease course of COVID-19, despite being in an era where specific vaccines are available for the same. The phenomenon of non-seroconverters and the rapidly mutating nature of the SARS-CoV-2 virus particle, the emergence of new variants, and the variable immunogenicity of the COVID-19 vaccines make this an attractive area for further research. Furthermore, the loss of routine vaccine coverage during the extensive and prolonged global lockdown prompts rigorous efforts for catch-up immunisation activities, particularly in developing nations, in light of the results of the current study.

It is worth noting that the study had some limitations. Firstly, the sample size was small, which may have limited the statistical power of the analysis and reduced the generalizability of findings. Secondly, the follow-up period was short, so it is unclear how long the observed immune response will last, and long-term follow-up would be needed to assess the durability of the humoral response. Additionally, the study did not assess the neutralising value of the antibodies, which would provide a more accurate measure of protection against COVID-19. The study primarily focused on asymptomatic and mild cases, with low numbers of moderate, severe, and critically ill patients, which may affect the ability to draw conclusions regarding the effect of MR vaccination on disease severity. Finally, the study did not differentiate between different strains of measles and rubella in different vaccines. Despite these limitations, the study provides valuable insight into the potential role of heterologous immunity conferred by MMR vaccination against COVID-19 and the dose-wise response of MMR-containing vaccines against COVID-19. Further studies are needed to confirm these findings and to explore the underlying mechanisms of heterologous immunity in greater detail. Strengths of the study include a prospective comparative design, clear participant selection criteria, use of reliable measurement tools, and follow-up with repeated measurements over time.

## Conclusions

In conclusion, our study provides evidence that a single dose of an MR-containing vaccine may enhance the humoral response to COVID-19. While we did not find a significant difference in disease severity between the vaccinated and unvaccinated groups, the potential of MR-containing vaccines in modulating the pathogenesis of COVID-19 cannot be ruled out. Further randomized trials involving larger populations are needed to validate our findings and explore the impact of MR vaccination on COVID-19 immunity. Our study adds to the existing literature on the potential role of heterologous immunity in the fight against COVID-19.

## Appendices

The datasets used and/or analysed during the current study can be requested from the corresponding author.

## References

[1] Naserghandi A, Saffarpour R, Allameh SF (2020). Exploring the causes of mild COVID-19 involvement in pediatric patients. New Microbes New Infect.

[2] Sharma D (2021). Repurposing of the childhood vaccines: could we train the immune system against the SARS-CoV-2. Expert Rev Vaccines.

[3] Anbarasu A, Ramaiah S, Livingstone P (2020). Vaccine repurposing approach for preventing COVID 19: can MMR vaccines reduce morbidity and mortality?. Hum Vaccin Immunother.

[4] Sidiq KR, Sabir DK, Ali SM, Kodzius R (2020). Does early childhood vaccination protect against COVID-19?. Front Mol Biosci.

[5] Aaby P, Samb B, Simondon F, Seck AM, Knudsen K, Whittle H (1995). Non-specific beneficial effect of measles immunisation: analysis of mortality studies from developing countries. BMJ.

[6] Goodridge HS, Ahmed SS, Curtis N (2016). Harnessing the beneficial heterologous effects of vaccination. Nat Rev Immunol.

[7] Mina MJ, Metcalf CJ, de Swart RL, Osterhaus AD, Grenfell BT (2015). Long-term measles-induced immunomodulation increases overall childhood infectious disease mortality. Science.

[8] (2023). Immunization coverage. https://www.who.int/news-room/fact-sheets/detail/immunization-coverage.

[9] Ashford JW, Gold JE, Huenergardt MA (2021). MMR vaccination: a potential strategy to reduce severity and mortality of COVID-19 illness. Am J Med.

[10] Larenas-Linnemann DE, Rodríguez-Monroy F (2021). Thirty-six COVID-19 cases preventively vaccinated with mumps-measles-rubella vaccine: all mild course. Allergy.

[11] (2023). Guidelines for management of COVID-19 in children (18th June, 2021). https://www.mohfw.gov.in/pdf/GuidelinesforManagementofCOVID19inCHILDREN18June2021final.pdf..

[12] Bayram Z, Musharrafieh U, Bizri AR (2022). Revisiting the potential role of BCG and MMR vaccines in COVID-19. Sci Prog.

[13] Hassani D, Amiri MM, Maghsood F (2021). Does prior immunization with measles, mumps, and rubella vaccines contribute to the antibody response to COVID-19 antigens?. Iran J Immunol.

[14] Kandeil A, Gomaa MR, El Taweel A (2020). Common childhood vaccines do not elicit a cross-reactive antibody response against SARS-CoV-2. PLoS One.

[15] Lundberg L, Bygdell M, Stukat von Feilitzen G, Woxenius S, Ohlsson C, Kindblom JM, Leach S (2021). Recent MMR vaccination in health care workers and Covid-19: a test negative case-control study. Vaccine.

[16] Gujar N, Tambe M, Parande M (2021). A case control study to assess effectiveness of measles containing vaccines in preventing severe acute respiratory syndrome coronavirus 2 (SARS-CoV-2) infection in children. Hum Vaccin Immunother.

[17] Liu W, Russell RM, Bibollet-Ruche F (2021). Predictors of nonseroconversion after SARS-CoV-2 infection. Emerg Infect Dis.

